# Sun Exposure Score and Vitamin D Levels in Moroccan Women of Childbearing Age

**DOI:** 10.3390/nu15030688

**Published:** 2023-01-29

**Authors:** Ilham Lhilali, Noura Zouine, Aziza Menouni, Lode Godderis, Marie-Paule Kestemont, Adil El Midaoui, Samir El Jaafari, Younes Filali-Zegzouti

**Affiliations:** 1 Cluster of Competence Environment and Health, Faculty of Sciences, Moulay Ismail University, Meknes 50000, Morocco; 2 Higher Institute of Nursing Professions and Health Techniques, Meknes 50000, Morocco; 3 Health and Environment Unit, Faculty of Medicine, KU Leuven, 3000 Leuven, Belgium; 4 IDEWE, External Service for Prevention and Protection at Work, 3001 Heverlee, Belgium; 5 Statistic Unit, Louvain School of Management, University Catholique of Louvain, 1348 Louvain-la-Neuve, Belgium; 6 Faculty of Sciences and Techniques Errachidia, Moulay Ismail University of Meknes, Errachidia 52000, Morocco; 7 Department of Pharmacology and Physiology, Faculty of Medicine, University of Montreal, Montreal, QC H3C 3J7, Canada; 8 BASE Laboratory, Faculty of Sciences and Techniques Errachidia, Moulay Ismail University, Meknes 50000, Morocco

**Keywords:** sun exposure score, vitamin D deficiency

## Abstract

Sunlight exposure is an essential source of vitamin D for many humans. However, hypovitaminosis D is a global public health problem. This study aimed to develop and validate a sun exposure score (SES) and correlate it with serum 25-hydroxyvitamin D levels in women of childbearing age. One hundred and sixty women aged 18 to 45 years residing in Meknes, Morocco, were included. A questionnaire estimating the sun exposure score and blood analysis of serum 25-OHD concentration were performed. The questionnaire’s reliability and construct validity were evaluated using Cronbach’s alpha and factor analysis. Spearman’s test was used to assess the correlation between SES and 25-OHD levels. The score’s reliability and construct validity were good, with Cronbach’s alpha values >0.70 and factorial saturation ranging from 0.696 to 0.948. Serum 25-OHD levels were significantly associated with the total sun exposure score, and all SES domains (Rho was 0.615 (*p* < 0.0001), 0.307 (*p* < 0.0001), 0.605 (*p* < 0.0001), and 0.424 (*p* < 0.0001) for total SES, indoor exposure domain, outdoor exposure domain, and sun protection practice domain, respectively). In addition, median 25-OHD levels increased significantly when sun exposure was changed from insufficient to sufficient (*p* < 0.0001). The results suggest that the sun exposure score could be used as a clinical tool to assess vitamin D levels in women of childbearing age.

## 1. Introduction

Hypovitaminosis D is a worldwide health problem affecting growth and development with insidious health consequences, including increased risk of musculoskeletal disorders, falls, osteoporosis-induced fractures, chronic inflammatory diseases, cardiovascular problems, increased risk of cancers, respiratory infections, type 1 and type 2 diabetes, autoimmune diseases, and neurological and psychiatric disorders [1,2,3,4,5,6,7,8,9,10,11]. Recently, vitamin D has been implicated in reproductive health and pregnancy outcomes [12,13]. Clinical studies have shown that vitamin D supplementation can lower androgen levels, lower anti-Mullerian hormone (AMH) levels [14,15], normalize the metabolic profile, and regularize periods in women with polycystic ovarian syndrome (PCOS) [16,17]. In fact, low serum 25(OH)D concentrations have been linked to problems of infertility [18,19], endometriosis [20], polycystic ovary syndrome (PCOS) [16,17,21,22], as well as adverse pregnancy outcomes, including spontaneous abortions [23], gestational diabetes [24,25], bacterial vaginosis [26], preeclampsia [27,28], neonatal hypocalcemia [29], prematurity, and low birth weight [30], as well as fetal and infant growth disorders [31].

The World Medical Association estimates that nearly one-third of the population has a low serum vitamin D concentration below 30 ng/mL [32]. In Morocco, vitamin D deficiency is widespread, with a prevalence of 76.6 to more than 90%, especially in women over 50 years of age [33,34,35], and is becoming increasingly common among women of reproductive age. A study in Marrakech showed that 100% of participants aged 18–45 years had hypovitaminosis D (<30 ng/mL) [36]. Similarly, a recent national nutrition survey (NNS 2019) published in 2022 found that 78% of women of childbearing age have 25(OH)D levels below 20 ng/mL [37]. This situation is confusing because Morocco is one of the sunniest countries in the world [38]. The explanation for this phenomenon may be the lack of sun exposure [33], increased rates of obesity [37], sedentary lifestyles [13], and restrictive clothing habits [39]. 

The skin, through solar radiation, provides up to 90% of the body’s vitamin D needs [40,41,42], as only a few foodstuffs contain it (e.g., oily fish, eggs, meat, offal, mushrooms), and dietary intake of this vitamin is generally insufficient to cover requirements [43,44]. Cutaneous exposure to ultraviolet radiation with a wavelength between 290 and 315 nm induces the transformation of epidermic 7-dehydrocholesterol (7-DHC) into previtamin D3, which undergoes isomerization, giving rise to cholecalciferol or vitamin D3 [45,46]. However, this process is conditioned by a complex set of variables, including the solar zenith angle (number of available UVB photons), timing and mode of exposure, skin pigment, aging, exposed skin surface, and sun protection habits (sunscreen, clothing).

Latitude, altitude, season, weather, or time of day significantly affect the solar zenith angle (SZA) and, consequently, the amount of cutaneous vitamin D synthesis [45,47,48,49,50,51]. Thus, above and below latitudes of about 33° to 35°, cutaneous synthesis of vitamin D3 is very low or absent for most of the winter. This is also why very little or no vitamin D is synthesized in the skin during sun exposure before 10 a.m. and after 3 p.m., even in summer [49,50,51,52].

Personal characteristics such as age and skin pigmentation are also limiting factors. Vitamin D synthesis decreases with increasing age due to a drop in 7−dehydrocholesterol levels and in changes in skin morphology, resulting in a 50% decrease in the formation of previtamin D3 in the elderly compared to the young [49,53,54]. In the case of skin pigmentation, darker skin has more melanin, acting as a filter for ultraviolet radiation (UVR); research shows that type II skin converts epidermal 7-DHC to provitamin D3 approximately 5–10 times more efficiently than pigmented type V skin [45,55,56,57]. 

Sun protection behavior, such as shading out from the sun, lack of physical activity in open environments [58], clothing type [59,60,61,62], and sunscreen use [49,61], can affect UVB-7-dehydrocholesterol interactions by blocking, absorbing, reflecting, or scattering incident UV radiation.

Previously, sun exposure was measured using dosimeters or a brief sunlight diary. However, these tools have some limitations. Dosimeters are prohibitively expensive and require sensitive equipment and expertise; moreover, these devices cannot record the use of certain sun protection behaviors. A questionnaire or self-reported measures assessing sun exposure found in the literature estimate the duration and frequency of outdoor sun exposure with little or no consideration of covariates that could influence vitamin D photosynthesis, such as skin phototype, exposed body surface, and sun protection practices (type of clothing, use of sunscreens, etc.). In addition, the rating scales generally used were Likert-type (e.g., never to always) or rating scales measuring the consistency of use (e.g., always, often, sometimes, rarely, never). To our knowledge, only one study has developed a scoring algorithm for estimating outdoor sun exposure [63], but no questionnaire has assessed the sun exposure of individuals inside their homes. Indeed, lying down in a place where the horizon is flat, one receives a much higher dose of UV than walking in the street, where the body is often in the shade, and only a tiny amount of UV radiation is accessible [50]. In this sense, the household is the best place where homeowners can uncover their bodies for personal comfort and expose themselves to adequate sunlight on the terrace, backyard, patio, balcony, or garden. In the literature, a small number of studies evaluating exposure to natural sunlight in the home, Reid et al. [64] and Okan et al. [65] found that 25(OH)D increased significantly in people who sat for 15 or 30 min a day under the verandah or garden of the nursing home. In Morocco, traditional houses have a design centered on a courtyard or patio that allows natural sunlight to pass through and a roof terrace that serves as a solarium for sunbathing [66,67]. In addition, several countries in the world, including Morocco, tend to adopt a bioclimatic architectural design of housing according to the local climate to maximize the sunlight hours of the buildings and ensure thermal comfort [68,69].

The objective of this study was to develop and validate a sun exposure scale to assess the numerous factors influencing indoor and outdoor sun exposure, as well as the different sun protection techniques used, and correlate it with 25(OH)D levels in women of childbearing age living in Meknes, Morocco.

## 2. Methods

**Figure 1:**
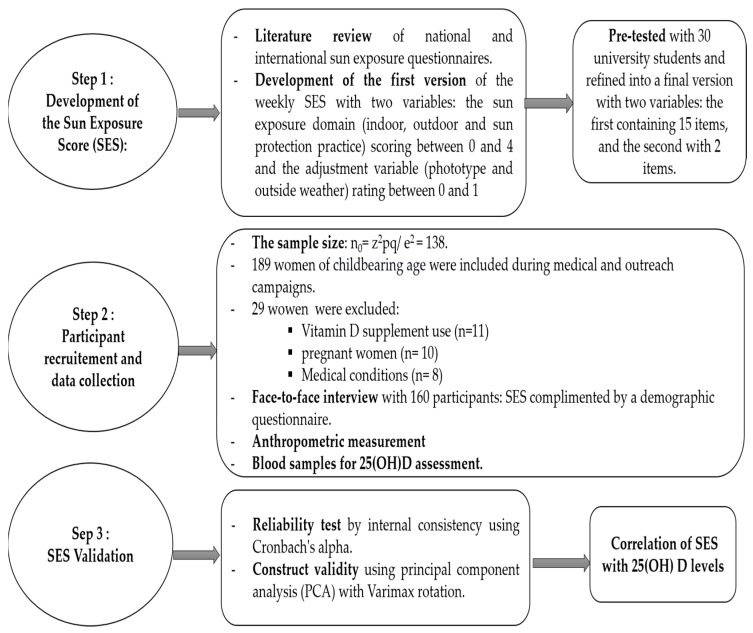
Steps in developing and validating sun exposure score.

The aim of this study was to develop and validate a score to measure sun exposure and correlate it with 25(OH)D levels. The methodology for developing and validating the score included three steps: (1) literature review and Sun Exposure Score (SES) development, (2) participant recruitment and data collection, and (3) construct validity and reliability of SES (Figure 1).

### 2.1. Step 1: Development of the Sun Exposure Score (SES)

#### 2.1.1. Literature Review and Sun Exposure Score (SES) Development

We developed a scale to estimate women’s sun exposure by considering all factors related to sun exposure that affect vitamin D levels, including phototype, weather, time and frequency of sun exposure, body parts exposed, and different sun protection behaviors. This score was developed from an extensive literature review of questionnaires assessing sun exposure in correlation or not with vitamin D levels [63,70,71,72,73,74,75,76,77]. A unique feature of our questionnaire is the inclusion of indoor exposure as a significant determinant of sun exposure, considering the specificity of Moroccan culture and home architecture that favor adequate indoor sun exposure.

This instrument is a questionnaire with two variables assessing weekly sun exposure: the first is composed of modifiable factors influencing sun exposure (duration, time slot, frequency of exposure, body part exposed, and sun protection behaviors). This variable contains 15 items, divided into three domains: indoor sun exposure, outdoor sun exposure, including work or routine activities, and outdoor recreation or leisure activities, and the third domain concerning sun protection practices. Each item is scored on a response option scale of 0 to 4 (e.g., for frequency of exposure: 0 if never; 1 if once/week; 2 if two to three times/week; 3 if four to five times/week; and 4 if more than five times/week). The second is an adjustment variable with two elements involving non-modifiable factors, the first being skin phototype based on Fitzpatrick’s skin type [78], namely: (I) white skin that always burns and never tans; (II) fair skin that burns easily and tans hardly; (III) fair to medium skin that burns moderately and always tans gradually; (IV) medium skin that burns little and tans easily; (V) brown skin that rarely burns and always tans darkly; and (VI) black, heavily pigmented skin that never burns and tans very easily. This item was scaled from 0 to 1 (0.25 if type V and VI, 0.5 if type III and VI, 0.75 if type II, and 1 if type I). The second item is the inside weather conditions, scored from 0 to 1 (0 if rainy, 0.25 if cloudy/lightly rainy, 0.5 if cloudy, 0.75 if sunny/lightly cloudy, and 1 if sunny).

The score attributed to each domain of the first variable corresponds to the sum of the scores of its different items multiplied by the scores of the second variable. The total score of sun exposure was classified according to four levels of satisfaction: insufficient, moderately sufficient, sufficient, and very sufficient or high. The sun exposure is considered insufficient if the total score is less than 7.5; moderate if it is between (7.5–15); sufficient if it is between (15–30); and very sufficient or high if it is greater than 30. Adequate sun exposure means sufficient scores in the indoor and outdoor SES domains and insufficient scores in the sun protection practices domain. Appendix A shows the different domains included in the score.

#### 2.1.2. Pretest of SES

A questionnaire pretest was conducted with 30 university students not included in the study to verify the questionnaire’s simplicity, fluidity, and comprehensibility. The questions were then readjusted based on the pretest results.

### 2.2. Step 2: Participant Recruitment and Data Collection

#### 2.2.1. Study Population

It is a cross-sectional study conducted in the prefecture of Meknes in north-central Morocco (coordinates 5° 32’ 26.628’’ W, 33° 52’ 22.8576’’ N, sea level), with an average daily maximum UV index in spring of 8, and an average of 08 h of sunlight per day in spring.

The sample size (n0) was set concerning Cochran’s formula [79]: n_0_ = z^2^pq/e^2^; where z is the value from the standard normal distribution reflecting the confidence level (Z = 1.96 for 95%); p is the estimated prevalence of hypovitaminosis D in Moroccan women of reproductive age (limited to studies defining hypovitaminosis D as a circulating level of 25(OH)D less than 30 ng/mL); p is calculated based on the mean prevalence of hypovitaminosis D in premenopausal and reproductive age women reported by Allali et al. [33] and Baki et al. [36], respectively, (*p* = 90%); q is 1 − p, and e is the desired level of precision set at 5%. Thus, 138 women were required to obtain statistically representative data.

A total of 160 healthy women of childbearing age (18 to 45 years) were included during medical and outreach campaigns conducted by the midwifery association in spring 2019. Exclusion criteria included participants with malabsorptive, hepatic, and renal conditions; pregnant or lactating women; vitamin D supplementation in the past three months; and women on corticosteroid therapy or other drugs known to alter vitamin D metabolism.

This study was conducted following the guidelines outlined in the Declaration of Helsinki, and all procedures involving human subjects were approved by the Ethics Committee for Biomedical Research of the Moulay Ismail University of Meknes (CERB-UMI protocol codes 201901 (January 2019)). All participants were recruited after obtaining written informed consent.

#### 2.2.2. Data Collection

The score questionnaire was conducted in a face-to-face interview and was complimented by a demographic questionnaire. In addition, a physical examination was performed to detect possible diseases that would prohibit participation in the study and to collect the participant’s medical history and anthropometric data. 

Serum 25-hydroxyvitamin D (25(OH)D) concentrations were determined from blood samples collected from the non-fasting participant in a red dry tube; the blood was centrifuged at 3000 rpm for 15 min at room temperature. Serum samples were immediately stored in aliquots at −80 °C until use. The serum samples were analyzed at the clinical laboratory of Mohamed V Hospital in Meknes, Morocco. Serum 25(OH)D determination was performed using an electrochemiluminescence (Cobas E411 immunoassay analyzer, Sterifil, casablaca, Morocco) measuring a range of 3–100 ng/mL or 7.5–250 nmol/L. Based on the thresholds of 25(OH)D levels proposed by the Research Group on Information on Osteoporosis (GRIO) and adopted by the Moroccan Society of Rheumatology to define a vitamin D deficiency or insufficiency, women were classified as sufficient (>30 ng/mL), insufficient (10 to 29.9 ng/mL), and deficient (<10 ng/mL) [80,81].

### 2.3. Step 3: SES Validation

#### Test of Construct Validity and Reliability of SES

The final questionnaire was interviewer-administered to the sample population. The reliability of the first variable of the questionnaire was assessed by internal consistency using Cronbach’s alpha. The scores below 5 were considered unacceptable, while those ranging from 0.50 to 0.59 were considered poor, 0.60 to 0.69 questionable, 0.70 to 0.79 acceptable, 0.80 to 0.89 good, and those >0.90 were rated as excellent [82,83].

The construct validity of the sun exposure questionnaire was assessed by adopting principal component analysis (PCA) with varimax rotation. The adequacy of factorial analysis models was checked using the Kaiser–Meyer–Olkin (KMO) test [84], and the Bartlett test for sphericity; the sampling was considered adequate at a KMO value greater than 0.6 and when Bartlett’s sphericity test was significant (*p* < 0.05). Based on the eigenvalues, total variance explained, and the scree plot, factors with eigenvalues >1 were selected, and a varimax rotation of the variance with Kaiser Normalization was performed. Thus, items with factor loadings values greater than or equal to 0.40 were grouped into a particular domain [85].

### 2.4. Statistical Analyses

The Shapiro–Wilk test was used to evaluate the normality of the quantitative data. Variables with a normal distribution were represented as mean values ± standard deviation (SD). For a non-normal distribution, data are expressed as median with an interquartile range (IQR). Categorical variables are represented as counts or frequencies. Kruskal-Wallis, one-way ANOVA, and chi-square tests for categorical and quantitative variables were used to compare participants’ demographic characteristics with the different domains and classes of the sun exposure score. The Spearman rank correlation coefficient was a non-parametric test to determine the association between SES scores and 25-OHD levels. Results were considered statistically significant at *p*-values < 0.05. All statistical analyses were realized using the statistical software R (version 4.0.4. R Foundation for Statistical Computing).

## 3. Results

A total of 160 women were included in the present study, with a median age of 25 years (18–45). A total of 63.7% were residents in urban areas and 58% lived in traditional houses, compared to 42% who lived in apartments. As for occupation, 50% of the participants were housewives, 35% were students, and 15% were officials/salaried. 

### 3.1. Validation of the Sun Exposure Score

As shown in Table 1, the KMO test = 0.755 is more significant than the minimum acceptable value of 0.6, and the Bartlett test shows a chi-square value of 1882.232 and *p* < 0.0001, indicating that there is sufficient correlation between the variables and that the data can be factorized. Thus, a principal component analysis with the varimax rotation method and Kaiser Normalization was performed to evaluate the construct validity of the sun exposure score.

**Figure 2:**
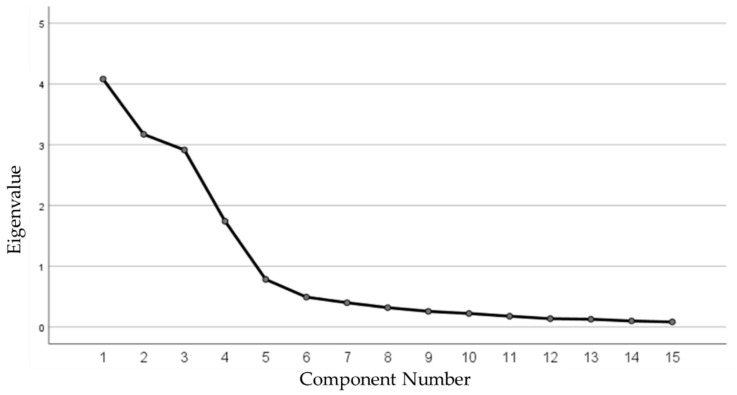
Scree plot by principal component analysis of the sun exposure score (SES).

The scree plot (Figure 2) indicates that four factors have an eigenvalue more significant than one and result in four common factors, which contribute to the cumulative variance of 79.37% of the 15 variables deemed sufficient in terms of total variance explained (Table 2). The four factors were entitled: (1) indoor sun exposure consisting of four items, (2) outdoor work or routine activities grouping four items, (3) outdoor recreational activities including four items, and (4) sun protection practices comprising three items. Regarding the validity or quality of the items that make up each factor, Table 2 shows that the 15 items have a saturation ranging from 0.699 to 0.948. 

The internal consistency coefficients for each of the four factors are, respectively, 0.909, 0.923, 0.835 and 0.787 (Table 2). The variables forming the same factor have good internal consistency since the minimum recommended value of Cronbach’s alpha is 0.70. This consistency in internal coherence is further evidence of the reproducibility of the results over time.

### 3.2. Participants’ Sun Exposure Score and Vitamin D Levels

**Figure 3:**
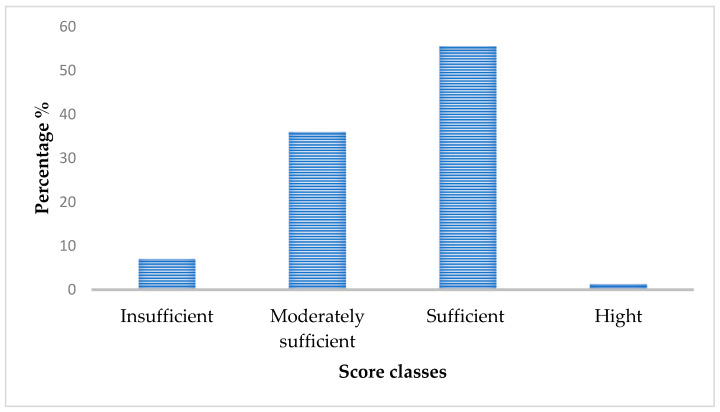
Sun exposure score classification of women of childbearing age.

Figure 3 shows that about 57% of the women surveyed had a sufficient score of sun exposure between 7.5–15; due to the smaller sample size of the high score (n = 2), they were fused with the sufficient score. In contrast, 36% and 7% of the participants had moderate and insufficient sun exposure, respectively.

As presented in Table 3, the mean ±SD exposure score was 15.82 ± 5.64. Primiparous women, officials/salaried women, participants living in rural areas, and participants residing in houses had the highest mean of SES (19.46, 18.02, 17.72, and 17.03, respectively). Participants with insufficient and moderate sun exposure were predominantly obese women, participants living in apartments, and students.

Table 4 indicates that women living in rural areas and houses had a higher median score for indoor exposure, which explains their sufficient exposure compared to those living in urban areas and apartments (*p* < 0.0001). Regarding outdoor exposure, officials/salaried women and those with a normal weight had a higher median score compared to others (10.96, *p* = 0.007 and 10.13, *p* < 0.0001, respectively).

Table 5 shows that the median [IQR] for indoor exposure, outdoor exposure, and sun protection practice SES domains were 4.78 [3.00], 7.88 [6.19], and 3.38 [3.18], respectively. Women with moderate scores were exposed more indoors than outdoors, whereas those with sufficient scores had higher outdoor than indoor scores. Women insufficiently sun exposed had higher sun protection practice scores than those sufficiently exposed to the sun.

As reported in Table 6, serum 25-OHD levels were significantly associated with the total sun exposure score and all SES domains (Spearman’s Rho was 0.615 (*p* < 0.0001), 0.307 (*p* < 0.0001), 0.605 (*p* < 0.0001), and 0.424 (*p* < 0.0001) for total SES, indoor exposure domain, outdoor exposure domain, and sun protection practice domain, respectively).

Table 7 reveals that all participants had hypovitaminosis D (<30 ng/mL), with a median of 8.42. Nevertheless, the medians of 25-OHD levels increased significantly when sun exposure progressed from insufficient to sufficient (4.78 and 10.47, respectively; *p* < 0.0001). In addition, most women (87%) with insufficient or moderately sufficient sun exposure had vitamin D deficiency (<10 ng/mL), whereas women with vitamin D insufficiency (10–30 ng/mL) had an adequate sun exposure score (19.88).

## 4. Discussion

Endogenous synthesis of vitamin D requires occasional exposure to UV radiation from the sun. Most experts consider the recommendation of daily exposure of 25% of the unprotected skin surface for 5 to 15 min between 10:00 a.m. and 3:00 p.m. in the fall, spring, and summer to be generally sufficient for people with skin types II or III. People with skin type V require more prolonged sun exposure of 25 to 40 min [45,47,55,56,57,86]. Such exposure increases vitamin D levels to a level semblable to an oral dose of 1000 IU [87,88].

This research is the first study to develop and validate a sun exposure score (SES) correlated with 25(OH)D levels for women of childbearing age in Meknes, Morocco. The questionnaire score was designed to estimate overall sun exposure according to skin phototype, and outside weather, encompassing indoor exposure, outdoor exposure, and habitual sun protection practices in the week prior to the survey. The one-week SES was adopted to provide participants with an accurate recall and account of sun exposure behavior. However, some studies have examined longer periods, ranging from a few weeks to several years, allowing a more reliable estimate of sun exposure for people who rarely expose themselves [63,71,76,77,89]. Furthermore, the SES was administered only once since exposure behavior constantly changes depending on heat preferences, sun protection measures [90], individual needs, and habits. In addition, leisure activities depend more on weather conditions such as temperature, humidity, and sun duration than routine activities [91].

Our results indicate that the SES is valid for classifying study participants according to their level of weekly sun exposure; this is based on the questionnaire’s good validity and reliability results. In addition, we found a significant association between the SES and serum 25 (OH) D concentrations (Rho = 0.615 (*p* < 0.0001).

The score questionnaires were evaluated for construct validity and reliability. The construct validity of our score questionnaire was satisfactory. Factor analysis identified a four-factor solution that accounted for 79.3% of the total variance, namely: indoor sun exposure, outdoor work or routine activities grouping, outdoor recreational activities including four items, and sun protection practices. All items had high loadings ranging from 0.699 to 0.948. This analysis indicated that no items needed to be excluded from our sun exposure score.

SES has also revealed good to excellent internal consistency for all four factors, illustrated by a high Cronbach’s alpha exceeding the required minimum threshold of 0.70 [82,83]. This high index suggests a substantial homogeneity of the items of the score [92]. However, very few studies on sun exposure have validated their measurement questionnaires. In China, a questionnaire on lifetime sun exposure revealed two factors with factor loadings ranging from 0.616 to 0.908 [76]. In Spain, a principal component analysis of the CHACES questionnaire on knowledge, attitudes, and habits related to sun exposure in young adults showed that two components accounted for more than 50% of the total variance. The reliability of the CHACES questionnaire was proven with a Cronbach’s coefficient between 0.45 and 0.8 for all components, except knowledge (0.335) and test-retest reliability (absolute agreement > 60%) [93]. Another questionnaire of the Sun Exposure Protection Index (SEPI), developed in Sweden and Australia, showed that an internal consistency as reflected by Cronbach’s alpha was found to be slightly below the desired value of >0.70 [71]. In contrast, Yu et al. [77] validated an urban Filipino adult sun exposure questionnaire (SEQ) by grouping items with similar factor loadings into three domains, even with a lower value than recommended for factor analysis (0.4) [85]. Furthermore, they based the validation of the SEQ on a sufficient content validity index of at least 0.86 and satisfactory internal consistency with an overall Cronbach alpha of 0.80.

Our results revealed sufficient sun exposure in 57% of the participants, with an average of 15.82. The median indoor exposure, outdoor exposure, and sun protection practices were 4.78, 7.88, and 3.38, respectively. Women insufficiently sun-exposed had higher sun protection practice scores than those sufficiently exposed to the sun. These results are consistent with other findings in the literature [39,63,94], in contrast to Abda et al. [95], who found that the prevalence of sun exposure is high in Morocco (52.1%) and that sun protection practices are often inadequate. 

Moreover, our SES showed that women living in rural areas and houses had a higher median score explained by high indoor sun exposure compared to those living in urban areas and/or apartments. In this context, the total sun exposure score of women inside their homes in the yard or on the terrace, especially in rural areas, may be more important than the self-reported frequency and time spent outdoors. This can also be explained by the fact that the outdoor activities of the rural participants begin early in the morning, before 10:00 a.m., whether it is raising livestock, collecting water, or gathering wood.

In our results, the sun exposure score was negatively correlated with BMI; obese women had a lower score than others, as did students, who had a lower mean score of 13.56 than housewives (16.73) and official/salaried women (18.02). Comparable results were reported by Dadda et al. [39] who showed that high vitamin D levels can be linked to outdoor activities with greater exposure to the sun.

Despite sufficient sun exposure in 57% of women, all participants had hypovitaminosis D < 30 ng/mL, with a median of 8.42 ng/mL. Baki et al. [36] and the national nutrition survey (ENN 2019) [37] confirms the high prevalence of hypovitaminosis D in women of childbearing age in Morocco. Nevertheless, the findings indicated a significant increase in 25-OHD levels as sun exposure progressed from insufficient to sufficient (4.78 and 10.47, respectively; *p* < 0.0001). Similar results were found by Mansibang et al. [94], who reported that the mean 25-OHD levels of the different groups increased significantly when sun exposure moved from low to high (*p* < 0.001). Humayun et al. [63] observed that the mean serum 25-OHD concentration was lower in all sun exposure groups. Similarly, in Morocco, the national nutrition survey (ENN 2019) [37] reported that the duration of exposure to sunlight has a large impact on vitamin D status, with significantly less vitamin D deficiency in women who were exposed to the sun for more than 20 min/d than those who were exposed to the sun for less than 20 min/d or who were not exposed to the sun (27.6%). In the same sense, Dadda et al. [39] explain the high prevalence of hypovitaminosis D (94%) in the Draa-Tafilalet region by restrictive clothing habits and low daily sun exposure.

In this study, serum 25-OHD levels were significantly associated with total sun exposure score and all SES domains (Rho = 0.615, 0.307, 0.605 and 0.424; *p* < 0.0001, for total SES, indoor exposure domain, outdoor exposure domain, and sun protection practice domain, respectively). The results of our study are consistent with previous research. Hanwell et al. [72] in Italy demonstrated a significant correlation between weekly sun exposure score and 25(OH)D concentrations during summer (Rho = 0.59, *p* = 0.003). Humayun et al. [63] in Pakistan showed a moderate correlation between self-reported SEQ and serum 25-OHD (short-term SEQ (Rho = 0.36, *p* = 0.01) and long-term SEQ (Rho = 0.43, *p* = 0.01)) in summers and 0,48 (*p* = 0,01) in winters. In Philippines, Yu et al. [77] reported a moderate correlation between 25-OHD levels and SEQ (r = 0.396, *p* < 0.001). Similarly, in Morocco, Dadda et al. [39] indicated a positive correlation of vitamin D levels with daily sun exposure (r = 0.308, *p* < 0.001). Conversely, in a study by Cargill et al. [70] in Australia and Sham et al. [96] in Canada, there was no significant correlation between questionnaire measures of time outdoors on weekdays, weekends, or cumulative time and 25-OHD concentration.

Our study showed that 43% of women of childbearing age had an insufficient to moderate sun exposure score, and all had a hypovitaminosis D below 30 ng/mL. This may be related to factors other than sun exposure that are not accounted for in the questionnaire score, such as pollution and daily dietary vitamin D intake. Another limitation was the short estimation period of sun exposure limited to one week, which could be more susceptible to reflect effects specific to the situation of rarely exposed women but could be counterproductive for those with frequent sun exposure. 

## 5. Conclusions

The main objective of this research is to adopt an appropriate and simple tool to measure sun exposure, which is an essential determinant of vitamin D levels. The SES developed in this study had good construct validity and reliability for estimating and classifying sun exposure in female participants of reproductive age. Furthermore, the SES showed a significant positive correlation with 25(OH)D levels. Therefore, it could be a valuable clinical tool to evaluate sun exposure and identify individuals at risk for vitamin D deficiency and associated diseases. However, we recognize that because of limitations in participant size and the spring study period, the predictive value of the current score needs to be validated in other experimental conditions with a larger population (women and men) at different latitudes/altitudes and seasons of the year.

## Figures and Tables

**Table 1:** Results of Kaiser–Meyer–Olkin (KMO) and Bartlett’s test.

KMO Measure of Sampling Adequacy		0.755
Bartlett’s Test of Sphericity	Approx. Chi-Square	1882.232
	Df	105
	Sig.	0.000

**Table 2:** Results of principal component analysis of the SES (varimax-rotated with Kaiser Normalization).

Item Code	Item Description	Component				Communalities
		1	2	3	4	
**Indoor sun exposure**						
**1**	Frequency of indoor sun exposure (in terrace, balcony, courtyard)	0.111	**0.842**	−0.146	0.141	**0.763**
**2**	Indoor exposed body part	0.012	**0.936**	−0.102	−0.011	**0.887**
**3**	Indoor duration sun exposure	0.004	**0.913**	0.017	0.021	**0.834**
**4**	Indoor sun exposure time slot	−0.050	**0.907**	−0.034	0.074	**0.832**
**Outdoor professional or routine activities**						
**5**	Frequency of professional or routine outdoor activities,	−0.059	−0.248	**0.746**	0.002	**0.622**
**6**	A time slot for professional or routine outdoor activities	−0.002	−0.044	**0.856**	0.039	**0.736**
**7**	Duration of professional or routine outdoor activities	−0.013	0.068	**0.895**	0.131	**0.824**
**8**	Outdoor exposed body part	−0.166	−0.046	**0.873**	−0.009	**0.792**
**Outdoor recreational activities**						
**9**	Practice outdoor activities (sports, hiking, walking, camping, swimming, etc.)	**0.896**	0.012	0.024	0.008	**0.803**
**10**	Duration outdoor activities	**0.922**	0.025	0.020	0.087	**0.858**
**11**	Time slot for sun exposure in the open air	**0.838**	0.008	−0.306	0.079	**0.802**
**12**	Part of the body exposed in the open air	**0.948**	0.026	−0.057	0.041	**0.904**
**Sun protection practices**						
**13**	Type of clothing usually used	−0.176	−0.041	0.004	**0.699**	**0.522**
**14**	Type of sunscreen used	0.211	0.126	0.041	**0.911**	**0.892**
**15**	Staying out of the sun	0.221	0.138	0.112	**0.868**	**0.834**
**Initial Eigenvalues**		4.081	3.170	2.914	1.741	
**Explained variance %.**		27.207	21.136	19.427	11.604	
**Cumulative variance %.**		27.207	48.343	67.770	**79.374**	
**Cronbach’s alpha coefficient**		**0.909**	**0.923**	**0.835**	**0.787**	

**Table 3:** Correlation of socio-demographic characteristics with sun exposure score classes.

Socio-Demographic Characteristics		Total (N = 160)	Sun Exposure Score		Sun Exposure Classes			
			(N = 160)	*p*-Value	Insufficient (N = 11)	Moderately Sufficient (N = 58)	Sufficient ^e^ (N = 91)	*p*-Value
			**Mean ± SD**		**Frequency (%)**			
**Age (yr)** (Median [IQR])		25 [11]	15.82 ± 5.64	0.211 **^a^**				0.858 **^b^**
	18–25	86 (53.7)	15.23 ± 5.70		6 (54.5)	34 (58.6)	46 (50.5)	
	26–35	54 (33.8)	17.05 ± 5.62		3 (27.3)	18 (31)	33 (36.3)	
	36–45	20 (12.5)	14.97 ± 5.09		2 (18.2)	6 (10.4)	12 (13.2)	
**BMI (kg/m^2^)** (Median [IQR])		25.70 [8.65]		**0.018** ^a^				**0.02 ^b^**
	Normal weight (<25 kg/m^2^)	68 (42.5)	16.31 ± 5.61		3 (27.3)	24 (41.4)	41 (45.1)	
	Overweight (25.0 to <30 kg/m^2^	44 (27.5)	16.93 ± 6.07		4 (36.4)	9 (15.5)	31 (34.1)	
	Obese (>30 kg/m^2^)	48 (30)	14.09 ± 4.94		4 (36.4)	25 (43.1)	19 (20.9)	
**Location living %**				**0.001** ^C^				
	Urban	102 (63.7)	14.73 ± 5.47		9 (81.8)	42 (72.4)	51 (56)	0.056 ^d^
	Rural	58 (36.3)	17.72 ± 5.38		2 (18.2)	16 (27.6)	40 (44)	
**Housing %**				**0.001** ^C^				**0.003** ^d^
	House	93 (58.1)	17.03 ± 5.65		6 (54.5)	24 (41.4)	63 (69.2)	
	Apartment	67 (41.9)	14.13 ± 5.21		5 (45.5)	34 (58.6)	28 (30.8)	
**Marital status %**				**<0.0001** ^C^				**0.002** ^d^
	Married	84 (52.5)	14.73 ± 5.47		4 (36.4)	21 (36.2)	59 (64.8)	
	Single	76 (47.5)	14.73 ± 5.47		7 (63.6)	37 (63.8)	76 (47.5)	
**Parity %**				**<0.0001** ^C^				**0.006** ^d^
	Nulliparous	76 (47.5)	14.05 ± 5.68		7 (36.6)	37 (63.8)	32 (35.2)	
	Primiparous	18 (11.3)	19.46 ± 5.78		1 (9.1)	2 (3.4)	15 (16.5)	
	Multiparous	66 (41.2)	16.84 ± 4.85		3 (27.3)	19 (32.8)	44 (48.4)	
**Profession %**				**<0.0001** ^C^				**0.033** ^d^
	Housewife	80 (50)	16.73 ± 5.58		6 (54.5)	22 (37.9)	52(57.1)	
	Students	56 (35)	13.56 ± 5.04		5 (45.5)	28 (48.3)	23 (25.3)	
	Official/salaried	24 (15)	18.02 ± 5.67		0	8 (13.8)	16 (17.6)	

^a^ Spearman test. ^b^ Kruskal –Wallis test. ^C^ Univariate ANOVA test. ^d^ Pearson’s chi-squared test. ^e^ Due to the small sample size of the high score (n = 2), they were merged with the sufficient score.

**Table 4:** Correlation of socio-demographic characteristics with sun exposure score domains.

Socio-Demographic Characteristics		Indoor Sun Exposure		Outdoor Sun Exposure		Sun Protection Practices	
		Median [IQR]	*p*-Value	Median [IQR]	*p*-Value	Median [IQR]	*p*-Value
**Age (yr)**			0.16 ^a^		0.633 ^a^		0.266 ^a^
	18–25	4.50 [4.5]		7.31 [6.19]		3.38 [3.52]	
	26–35	5.06 [2.81]		9.56 [6.75]		3.38 [2.95]	
	36–45	4.5 [4.22]		7.31 [6.47]		3.38 [1.64]	
**BMI (Kg/m^2^)**			0.152 ^a^		**<0.0001** ^a^		0.673 ^a^
	Normal weight (<25 kg/m^2^)	4.50 [5.06]		10.13 [6.19]		3.38 [3.94]	
	Overweight (25.0 to <30 kg/m^2^)	5.16 [2.16]		8.34 [5.91]		3.38 [2.06]	
	Obese(>30 kg/m^2^)	4.78 [3.33]		6.28 [3.38]		3.38 [2.67]	
**Location living**			**<0.0001** ^b^		0.907 ^b^		**0.001** ^b^
	Urban	3.75 [5.06]		7.41 [5.63]		3.56 [2.95]	
	Rural	5.63 [1.69]		8.91 [6.75]		3.38 [2.06]	
**Housing**			**<0.0001** ^b^		0.356 ^b^		**0.007** ^b^
	House	5.06 [1.41]		7.88 [6.75]		3.38 [3.38]	
	Apartment	1.5 [4.5]		7.88 [5.63]		3.38 [2.06]	
**Marital status**			**<0.0001** ^b^		0.309 ^b^		**<0.0001** ^b^
	Married	5.06 [1.13]		9.56 [6.19]		3.38 [3.38]	
	Single	3.38 [5.06]		7.31 [5.90]		3.38 [1.69]	
**Parity**			**<0.0001** ^b^		0.14 ^b^		**0.001** ^b^
	Nulliparous	3.38 [5.06]		7.31 [5.90]		3.38 [1.68]	
	Primiparous	5.63 [1.26]		10.97 [5.06]		3.38 [2.25]	
	Multiparous	5.06 [1.73]		7.59 [6.75]		3.38 [3.38]	
**Profession**			**<0.0001** ^b^		**0.007** ^b^		**0.003** ^b^
	Housewife	5.06 [1.5]		7.41 [6.61]		3.38 [3.38]	
	Students	3.38 [5.06]		7.31 [5.34]		3.09 [1.69]	
	Official/salaried	4.78 [5.48]		10.69 [5.48]		3.38 [1.36]	

^a^ Spearman test. ^b^ Univariate ANOVA test.

**Table 5:** Sun exposure scores by domains.

SES Domains	Sun Exposure Classes						
	Total Median [IQR]	Spearman’s Rho	*p*-Value ^a^	Insufficient	Moderately Sufficient	Sufficient	*p*-Value ^b^
				Median [IQR]			
Indoor sun exposure	4.78 [3.00]	0.65	**<0.0001**	1.5 [2.06]	6.80 [4.84]	10.47 [6.66]	**<0.0001**
Outdoor sun exposure	7.88 [6.19]	0.827	**<0.0001**	2.48 [1.31]	6.09 [2.25]	11.25 [4.5]	**<0.0001**
Sun protection practices	3.38 [3.18]	0.658	<0.0001	1.13 [0.75]	2.25 [1.69]	3.75 [2.25]	**<0.0001**
Pairwise comparisons of sun exposure classes							
Insufficient-moderately sufficient			0.103				
Insufficient-sufficient			**<0.0001**				
Moderately sufficient-sufficient			**<0.0001**				

^a^ Spearman test. ^b^ Kruskal–Wallis test.

**Table 6:** Correlation of sun exposure scores domains with 25-OHD Levels.

SES Domains	Serum 25-OHD (ng/mL)	
	Spearman’s Rho	*p*-Value
Sun exposure score	0.615	**<0.0001**
Indoor sun exposure	0.307	**<0.0001**
Outdoor sun exposure	0.605	**<0.0001**
Sun protection practices	0.424	**<0.0001**

**Table 7:** Participants’ vitamin D levels were ranked according to sun exposure score classes.

Sun Exposure Classes	Serum 25-OHD (ng/mL)		Vitamin D Status		
	N = 160 Median [IQR]	*p*-Value ^a^	Deficiency	Insufficiency	*p*-Value
			N = 104	N = 56	
			Frequency (%)		
Insufficient	4.78 [4.65]	**<0.0001**	11 (10.6)	**0**	**0.001 ^b^**
Moderately sufficient	6.80 [4.84]	**<0.0001**	49 (47.1)	9 (16.1)	
Sufficient	10.47 [6.66]	**<0.0001**	44 (42.3)	47 (83.9)	
Sun exposure score **(Mean ± SD)**	8.42 [6.41]	**<0.0001**	13.62 ± 4.81	19.88 ± 4.78	**<0.0001 ^c^**

^a^ Spearman test. ^b^ Pearson’s chi-squared test. ^c^ Univariate ANOVA test.

## Data Availability

The data used to support the findings of this study are available from Prof. Filali-Zegzouti Younes upon request.

## Appendix A

**Sun Exposure Score (SES)**						
**Variable Sun Exposure Domains:**						
	**Items:**	**0**	**1**	**2**	**3**	**4**
Indoor sun exposure	Frequency of indoor sun exposure (in terrace, balcony, courtyard)	Never	1 time/week	2 to 3 times/week	4 to 5 times/week	More than 5 times/week
	Indoor exposed body part		Face	Face, hand	Face, arm	Face, arm, leg
	Indoor duration sun exposure	Less than 5 min	5 to 15 min	15 to 30 min	30 to 60 min	More than 60 min
	Indoor sun exposure time slot	Before 7 a.m.	7 a.m. to 9 a.m.	9 a.m. to 11 a.m./5 p.m. to 19 p.m.	11 a.m. to 1 p.m.	1 p.m. to 5 p.m.
Outdoor sun exposure	Frequency of professional or routine outdoor activities	Never	1 time/week	2 to 3 times/week	4 to 5 times/week	More than 5 times/week
	A time slot for professional or routine outdoor activities	Before 7 a.m.	7 a.m. to 9 a.m.	9 a.m. to 11 a.m./5 p.m. to 19 p.m.	11 a.m. to 1 p.m.	1 p.m. to 5 p.m.
	Duration of professional or routine outdoor activities	Less than 5 min	5 to 15 min	15 to 30 min	30 to 60 min	More than 60 min
	Outdoor exposed body part		Face	Face, hand	Face, arm	Face, arm, leg
	Practice outdoor activities (sports, hiking, walking, camping, swimming, etc.)	Never	1 time/week	2 to 3 times/week	4 to 5 times week	More than 5 times/week
	Duration outdoor activities	Less than 5 min	5 to 15 min	15 to 30 min	30 to 60 min	More than 60 min
	Time slot for sun exposure in the open air	Before 7 a.m.	7 a.m. to 9 a.m.	9 a.m. to 11 a.m./5 p.m. to 19 p.m.	11 a.m. to 1 p.m.	1 p.m. to 5 p.m.
	Part of the body exposed in the open air		Face	Face, hand	Face, arm	Face, arm, leg
Sun protection practices	Staying out of the sun	Used shade of tree/building/ parasol etc.	Market in the shade	Car/bus/van smoked window/of	Car/bus/van glass windows up	No protection
	Type of sunscreen used	SPF sunscreen of 50 and +	SPF sunscreen from 30 to 50	Sunscreen SPF from 15 to 30	Other creams and lotions without knowledge of the FPS or SPF 6 to 10	No sunscreen
	Type of clothing usually used	Usual clothes with long sleeves/long pants/heavy/tight clothes with dark colorants	Usual clothes with long sleeves/long pants/loose with dark colorants	Long loose clothing with light coloring.	Short sleeve/pants/shorts	Short no shirt /bikini
**Adjustment variable**						
	**Items:**	**0**	**0.25**	**0.5**	**0.75**	**1**
	Phototype		Type V & VI	Type III & IV	Type II	Type I
	Weather outside	rainy	cloudy/light rainy	cloudy	sunny/light cloudy	sunny
Total	Sun exposure score = the sum of the domain scores x phototype score x outdoor weather score.					
The score is considered insufficient if the score is below 7.5, moderate if the score is between 7.5–15, sufficient if the score is between 15–30, and as very sufficient or high if the score is above 30. Indoor sun exposure is considered insufficient if the score is below 4, moderately sufficient if the score is between 4 and 8, sufficient if the score is between 8 and 12, and high for a total above 12. For outdoor sun exposure, it is considered insufficient if the total is less than 8, moderately sufficient if the total is between 8 and 16, sufficient if the score is between 16 and 24, and high for a total greater than 24. Finally, for sun protection practices, this subscale has an inverse relationship with sun exposure: a score below 3 is considered high and considered sufficient from 3 to 6, moderately sufficient if the score is between 6 and 9, and insufficient sun protection practices for a score between 9 and 12.						
